# Vaccination day and perceived stress among university teachers

**DOI:** 10.1192/j.eurpsy.2022.1388

**Published:** 2022-09-01

**Authors:** A. Haddar, I. Sellami, A. Abbes, N. Derbel, H. Halweni, R. Masmoudi, M. Bouattour, J. Masmoudi, K. Hammami, M.L. Masmoudi, M. Hajjaji

**Affiliations:** 1 Hedi Chaker university hospital, Occupational Medicine, Sfax, Tunisia; 2 medecine university of Sfax, General Practice, Sfax, Tunisia; 3 HEDI CHAKER hospital, Psychiatry Department, SFAX, Tunisia; 4 Hospital university of HEDI CHAKER, Psychiatry A Department, Sfax, Tunisia

**Keywords:** Covid-19, Perceived stress, vaccination

## Abstract

**Introduction:**

Covid-19 vaccination in adults become a common behaviour nowadays. It may induce stress in some of the vaccinated patients.

**Objectives:**

This study aimed to evaluate perceived Stress among university teachers desiring to be vaccinated.

**Methods:**

We conducted a cross-sectional study on Tunisian university teachers who participated in a COVID-19 vaccination campaign organized in June 2021. A self-administered questionnaire was administered. The survey dealt with socio-professional data and the level of stress assessed with the Perceived Stress Scale (PSS-10).

**Results:**

A total of 100 participants were included. The mean age was 51 years ±7. The Sex Ratio (M/F) was 1.7. The majority of participants were married and reported living with their families (96%). On a 0 to 10 scale, 71% of participants described an excellent health status and rated it greater than or equal to 8. The average job tenure was 15 years. The PSS-10 showed moderate and high perceived stress in 86% and 4% of participants, respectively. Only 10% of university teachers presented low-stress perception.

**Conclusions:**

Getting vaccinated against Covid-19 is crucial in order to protect the population. This behaviour could be associated with a big amount of stress. Taking into account the psychiatric mental condition is crucial for the vaccinating health care providers in order to alleviate this experience.

**Disclosure:**

No significant relationships.

